# New Developments in the Classification, Pathogenesis, Risk Factors, Natural History, and Treatment of Branch Retinal Vein Occlusion

**DOI:** 10.1155/2017/4936924

**Published:** 2017-03-12

**Authors:** Jia Li, Yannis M. Paulus, Yuanlu Shuai, Wangyi Fang, Qinghuai Liu, Songtao Yuan

**Affiliations:** ^1^Department of Ophthalmology, The First Affiliated Hospital of Nanjing Medical University, Nanjing, China; ^2^Department of Biomedical Engineering, University of Michigan, Ann Arbor, MI, USA; ^3^Department of Ophthalmology and Visual Sciences, University of Michigan, Ann Arbor, MI, USA

## Abstract

For years, branch retinal vein occlusion is still a controversial disease in many aspects. An increasing amount of data is available regarding classification, pathogenesis, risk factors, natural history, and therapy of branch retinal vein occlusion. Some of the conclusions may even change our impression of branch retinal vein occlusion. It will be beneficial for our doctors to get a deeper understanding of this disease and improve the treatment skills. The aims of this review is to collect the information above and report new ideas especially from the past a few years.

## 1. Introduction

Retinal vein occlusion (RVO) is the second most common type of retinal vascular disorder, after diabetic retinal disease, and one of the most common causes of the sudden painless unilateral loss of vision [1]. RVO can be divided into two main types: branch retinal vein occlusion (BRVO) and central retina vein occlusion (CRVO). The International Eye Disease Consortium reported the prevalence of retinal vein occlusion in the USA, Europe, Asia, and Australia which contained 68751 individuals aging from 31 to 101 years of 15 studies which shows that the prevalence of RVO was 5.20 per 1000 for any RVO, 4.42 per 1000 for BRVO, and 0.80 per 1000 for CRVO. It suggested that roughly 16 million people in the world suffer from this vascular disorder and BRVO is about 4 times more common [2]. Generally, BRVO has a better prognosis than CRVO. Without therapeutic intervention, visual acuity can still improve generally in eyes with BRVO but clinically significant improvement beyond 20/40 was uncommon [3].

## 2. Classification

BRVO can be divided into two different types according to Hayreh et al.: major BRVO, when one of the major branch retinal vein is occluded, and macular BRVO, when one of the macular venules is occluded, and these two types have different fundus changes [4, 5]. Major BRVO comprises a nonischemic form and an ischemic form detectable in one third and two thirds of cases, respectively, and ocular neovascularization can only be found in ischemic major BRVO [6]. The typical arteriovenous crossing of major BRVO is situated along the course of a major venous branch. The location of the arteriovenous crossing with respect to the optic disc determines the extension of the area involved [7]. It is reported that 65% of BRVO occurred in the superior temporal quadrant. This is postulated to be due to increased arteriovenous crossing at this site or increased symptoms when the fovea becomes involved to affect the vision [8, 9]. Macular BRVO represents a particular venous occlusion in which the obstruction is limited to a small vein draining a specific sector of the macula located between the superior and inferior temporal arcades [10]. Unlike ischemic major BRVO, macular BRVO does not develop retinal neovascularization (NV) because the ischemic area is too small to provide a sufficient stimulus for NV. A further group is hemi-vein occlusion, a distinct clinical entity presenting as occlusion of only one trunk of the central retinal vein in the area of the anterior part of the optic nerve. Hayreh et al. considered it as a separate type since its pathogenesis is quite similar to CRVO [10]. With the help of basic technology such as fundus photography, fundus fluorescein angiography (FFA), spectral domain optical coherence tomography (SD-OCT), visual field testing, and full-field electroretinogram (ff-ERG), BRVO and macular edema are usually easy to diagnose and classify [11–14]. In addition, new technology such as OCT angiography (OCTA) can measure vascular density and the foveal avascular zone. OCTA can also observe the superficial and deep capillary networks, nonflow areas, vascular dilation, and intraretinal edema, which can be helpful in diagnosis and follow-up [15–19].

## 3. Pathogenesis

The pathogenesis of RVO is multifactorial while BRVO may be due to a combination of three primary mechanisms: compression of the vein at the arteriovenous (A/V) crossing, degenerative changes of the vessel wall, and abnormal hematological factors. Arteriolar sclerosis due to various reasons such as hypertension or hyperlipidemia can result in more compression of the veins, which is the primary cause of BRVO. Zhao et al. evaluated the anatomic position of the crossing vessels in 106 eyes with BRVO and found the artery anterior to the vein at the obstructed site in 99% of affected eyes [20]. Also, the mechanical obstruction of the vein through the rigid artery in the A/V crossing may result in turbulent blood flow producing damage to the vein vascular endothelium and intima media and the sequence of events leading to occlusion of the vein, as reported by Christoffersen and Larsen [21]. Hyperviscosity due to high hematocrit has also been found to be associated with BRVO [22]. Viscosity is mainly dependent upon the hematocrit and plasma fibrinogen, and isovolumetric hemodilution does also positively impact it [23]. Another discussed hematological disorder in the pathogenesis of BRVO is the dysregulation of the thrombosis-fibrinolysis balance. The coagulation cascade including different blood factors results in the production of thrombin which converts circulating fibrinogen to fibrin. The coagulation sequence is held in check and inhibited by specific anticoagulants including protein C, protein S, and antithrombin. However, the results of published studies are inconsistent, and the role of coagulation factors in the development of RVO remains unclear [24]. It is well recognized that submacular hemorrhage from neovascular age-related macular degeneration (AMD) or retinal artery macroaneuryms (RAMs) often causes severe visual impairment [25, 26]. Previous experimental studies have suggested several mechanisms by which subretinal hemorrhage damages the overlying photoreceptors, such as clot retraction, iron toxicity (hemosiderosis), induction of fibrosis, and blockage of nutrient diffusion from the choroidal circulation [27]. In BRVO, some of these mechanisms may be involved in the formation of foveal damage, although the mechanisms involved are not completely understood. An experiment carried out recently indicated that BRVO can cause acute endothelial cell apoptosis and increased permeability. Subsequently, the upstream vascular network remains destabilized, characterized by pericyte dropout, abnormally high endothelial cell turnover, and sensitivity to hypoxia. These early changes might pave the way for capillary loss and subsequent chronic ischemia and edema that characterize the late stage disease [28].

## 4. Cystoid Macular Edema

Cystoid macular edema (CME) is the main cause of impaired vision due to BRVO and occurs in 30% of BRVO eyes [29]. It was hypothesized to be caused by fluid flux from vessels to tissue according to Starling's law, which is based on the breakdown of the blood-retinal barrier as a result of damage to the tight junctions of capillary endothelial cells, vitreoretinal adhesion, and secretion into the vitreous of vasopermeability factors produced in the retina [30–33]. Noma et al. suggested that in patients with BRVO, vascular occlusion induces the expression of vascular endothelial growth factor (VEGF) which is promoted by hypoxia and retinal nonperfusion and interleukin-6 (IL-6), resulting in blood-retinal barrier (BRB) breakdown and increased vascular permeability which results in macular edema [34–36]. Moreover, aqueous levels of other growth factors such as placental growth factor (PIGF), platelet-derived growth factor (PDGF)-AA, and various inflammatory factors including soluble intercellular adhesion molecule (sICAM-1), monocyte chemoattractant protein (MCP-1), interleukin (IL)-6, IL-8, IL-12, and IL-13 as well as soluble vascular endothelial growth factor receptor (sVEGFR)-1 and sVEGFR-2 were found to be significantly higher and correlated with CME [36–38]. The inflammatory factors may induce an increase of vascular permeability and disrupt the blood-aqueous barrier, but further studies are needed to elucidate the exact pathophysiology [39]. Recently, a study reported that aqueous angiopoietin-like 4 (ANGPTL4) level in patients with ME due to BRVO is also significantly higher [40]. The ANGPLTL4 mediates the development of vascular permeability and angiogenesis in hypoxic conditions, is overexpressed in general ischemic retinopathy, and promotes the development of CME [41, 42]. If marked hypoxia persists, irreversible structural changes in the macular occur, and the disturbed visual acuity (VA) is almost always lasting. Moreover, it is recently reported that aqueous erythropoietin (EPO) level is also higher than normal in BRVO patients, especially during the acute period [43]. The EPO was found to be associated with retinal ischemia and provides neuroprotective effects against ischemia-reperfusion injury and light-induced retinal degeneration in animal models [44, 45]. It was discovered that there exists a strong correlation between EPO and VEGF. Higher concentrations of vitreous EPO in BRVO are exclusively caused by the retinal hypoxia and are related to CME [46].

## 5. Risk Factors

BRVO has many known ophthalmic and systemic risk factors considering its complicated pathogenesis. It is widely known that advancing age is an important risk factor for BRVO since the main pathogenic mechanism of BRVO is arterial stiffness that causes venous compression in the common adventitial sheath [2, 47]. Systemic vascular diseases like hypertension (HTN), hyperlipidemia (HLD), and peripheral arterial disease (PAD) and metabolic diseases like diabetes mellitus (DM) are all connected with BRVO. A meta-analysis showed that, in BRVO, the odds ratio for HTN, HLD, and DM is 3.0, 2.3, and 1.1, respectively [48]. A study in 2014 enrolled 492488 patients older than 55 years demonstrated that HTN seems to be the main driver of the increased risk for incident BRVO and those with more severe HTN were at even greater risk. Also, they discovered that persons with “uncomplicated” DM had no difference in the risk of being diagnosed with BRVO, while those with end organ damage from DM had a 36% increased risk, but no association between dyslipidemia and BRVO was found. However, the presence of dyslipidemia actually attenuated some of the increased risk of BRVO among persons who had multiple components of the metabolic syndrome [49]. Also Lam et al. reported that risk factors for developing BRVO younger than 50 years were very similar to those in older people (HTN, HLD, and high body mass index (BMI)); therefore, these two articles together can make a whole story [50]. It is not completely clear what role thrombophilia plays in the BRVO pathogenesis. Blood abnormalities play a controversial role in the pathogenesis of BRVO, and erythrocyte volume, level of fibrinogen, and hematocrit appear to be important [22–24]. Three recent meta-analyses of RVO and thrombophilic factors demonstrated that only hyperhomocysteinemia and anticardiolipin antibodies play a role in the pathogenesis of RVO [24, 29, 51]. Additional studies also reported other less common risk factors such as obstructive sleep apnea (OSA), inadvertent retrobulbar needle perforation, axial length and vitreous chamber depth, liver or renal diseases, and even posterior vitreous adhesion [52–57]. Moreover, BRVO may also be easier to find in people with gene defects, like adiponectin +276 G/T and AGTR1 A1166C single-nucleotide polymorphism, which are related to arterial stiffness [58]. Blacks were also found to be at increased risk of developing incident ophthalmic venous occlusive disease, even after controlling for HTN, other components of the metabolic syndrome, and sociodemographic factors [49]. These results support earlier work by the International Eye Disease Consortium, which showed a greater prevalence of BRVO among blacks compared with whites [2]. There are many hypotheses as to why blacks may be at greater risk for end organ vascular damage, ranging from reduced access to high-quality health care, to racism potentially leading to chronic stress, to living in neighborhoods with higher levels of pollution, and to living in unsafe neighborhoods impacting their ability to exercise, which can all lead to an increased burden of vascular disease [2].

## 6. Natural History

BRVO has a relatively better vision outcome compared to CRVO, with 50% to 60% of eyes recovering vision to 20/40 or better without treatment and with 25% which may develop retinal neovascularization [6]. The natural course of BRVO is determined by the site and degree of occlusion, the integrity of arterial perfusion to the affected sector, and the efficiency of the developing collateral circulation [59]. According to Hayreh et al., the median time to macular edema resolution was 21 months in those with major BRVO and 18 months in those with macular BRVO. Overall, for eyes with initial VA of 20/60 or better, VA improved or remained stable in 75% for major BRVO and 86% for macular BRVO. In those with initial VA of 20/70 or worse, VA improved in 69% for major BRVO and in 53% for macular BRVO, with median final VA of 20/60 for both BRVO types [60]. On follow-up, in temporal main BRVO, visual field defect improved or remained stable in 68% of eyes with minimal to mild initial defect and improved in 52% of eyes with moderate to severe initial defect. In macular BRVO, visual field defect remained stable or improved in 85% of eyes with minimal to mild initial defect [9]. A meta-analysis reported that 10% of the patients can observe the development of fellow eye involvement [3]. Although a majority of BRVO eyes had variable amounts of VA improvement without treatment, there was a lack of improvement in some eyes, which may be due to the same factors as those seen in ischemic central retinal vein occlusion: ischemic damage to macular retinal ganglion cells, pigmentary degeneration, and development of an epiretinal membrane from prolonged macular edema [5, 61–65].

## 7. Treatment

The treatment of BRVO is comprised of three main stages: identification and treatment of modifiable risk factors, specific treatment of the vascular occlusion, and treatment of BRVO complications such as macular edema, retinal neovascularization, vitreous hemorrhage, and traction retinal detachment so as to improve visual acuity and metamorphopsia [66]. The main purpose of all treatments is the resolution of the macular edema (the leading cause of impaired visual acuity and metamorphopsia) before the foveal photoreceptor layer is damaged [67, 68]. The management of macular edema secondary to branch retinal vein occlusion has greatly improved in recent times with the introduction of a therapy based on intravitreal injection of antivascular endothelial growth factor (VEGF) molecules and steroids [69]. Patient outcomes even with identical treatments can be vastly different due to disease, and patient heterogeneity prognostic factors for BRVO include patient age [70], baseline visual acuity and retinal thickness [51, 71, 72], early response to treatment [70], duration of macular edema [73, 74], posterior vitreous detachment [75], OCT characteristic [76–78], cytokine level [34, 79], central retinal sensitivity [80], leaking capillaries and microaneurysms in the perifoveal capillary network [60, 81, 82], retinal pigment epithelium (RPE) integrity [83, 84], serious retinal detachment [85], and subretinal hemorrhage [86]. Some of these prognostic factors are still controversial. Basically, patients with a younger age, milder symptom (such as nonischemic BRVO), shorter duration of CME, and better response to early treatment tend to have a better outcome. A recent survey reported that retina specialists treating CME secondary to RVO recommend different treatments for patients than they would choose for themselves. This suggests that cognitive biases exist and one should take this into consideration when making treatment recommendations for their patients [87].

### 7.1. Anti-VEGF

Inhibitors of vascular endothelial growth factor (VEGF) have revolutionized the treatment of CME associated with BRVO, a condition that is sensitive to VEGF. Several lines of evidence suggest that VEGF is a major mediator for CME in BRVO [33, 34] and have demonstrated the resolution of CME and improvement of vision in response to pharmacologic VEGF inhibition [88]. The most commonly used anti-VEGF drugs at this time are bevacizumab (Avastin), aflibercept (Eylea), and ranibizumab (Lucentis). Selected clinical trials are collected in Table 1 [89–99].

Bevacizumab is a recombinant humanized and chimeric IgG1 type monoclonal antibody, directed against all the isoforms of the VEGF peptide to block angiogenesis. Many studies have reported that visual acuity and macular edema improved significantly after intravitreal bevacizumab (IVB) treatment and also cause a significant decrease in sVEGFR-1, VEGF, PDGF-AA, MCP-1, and IL-8 [38, 89]. A recent study showed that after IVB treatment, there is an increase of retinal venous outflow that may possibly influence the resolution of macular edema [100]. Compared with intravitreal triamcinolone acetonide, intravitreal bevacizumab can achieve better long-term VA outcomes with much lower rate of adverse events (e.g., cataract and glaucoma), despite the fact that triamcinolone acetonide may achieve equal visual acuity and morphology improvement for the first few months right after treatment [101, 102]. Also, IVB can result in better outcome in the recurrent CME, while subthreshold grid laser was completely ineffective [103]. However, CME due to BRVO has a relatively high rate of recurrence. Only 30%–34% of IVB-effective eyes can achieve persistent resolution of CME, whereas most need additional treatments to optimize visual acuity [104–106]. The duration from symptom onset to initial IVB could affect the CME recurrence rate but not the efficacy rate after a single IVB injection [104].

Aflibercept is a fusion protein that combines key domains from human VEGF receptors VEGFRs-1 and VEGFRs-2 with the constant region Fc of human immunoglobulin G and binds multiple VEGF-A isoforms. The VIBRANT study (*n* = 183) included patients from North America and Japan and showed that 52.7% of the affected eyes had a visual improvement of more than 15 Early Treatment of Diabetic Retinopathy Study (ETDRS) letters with a mean improvement of 17 ETDRS letters. It also recommended a treatment mode of intravitreal aflibercept (IVA) every 8 weeks after a 24-week period of IVA every 4 weeks to obtain an optimal outcome at 52 weeks [91].

Ranibizumab is a Fab fragment that specifically binds all isoforms of vascular endothelial growth factor A. Both the BRAVO study (*n* = 397) and the BRIGHTER study (*n* = 455) demonstrated with ranibizumab a statistically significant superior improvement in best-corrected visual acuity (BCVA) compared with laser alone in patients with BRVO. No ocular side adverse effects (SAEs) were reported in this group [90, 107]. Although many studies claimed that intravitreal ranibizumab (IVR) can improve retinal nonperfusion, the effect was usually limited [108]. As discussed above, patients treated with IVR often have CME recurrence and needed frequent additional injections to cause complete CME resolution [95]. It is reported that four years after initial treatment, half of the patients still require treatment, but most of the outcomes were excellent [97]. In the natural course of acute BRVO, intraretinal hemorrhage and CME are absorbed gradually. However, IVR accelerates the speed of absorption of intraretinal hemorrhage and is associated with a rapid reduction of CME. Because there has been no evidence that anti-VEGF drugs modify the function of macrophages and microglia which can phagocytose debris and red blood cells, one can only postulate that ranibizumab does not facilitate the absorption of intraretinal hemorrhage directly but rather suppresses new bleeding and thus seemingly accelerates absorption [109].

Recently, conbercept (KH902), a recombinant and soluble VEGF receptor fused to the Fc portion of human IgG1 with 100% human protein sequence, has been developed. Because the addition of the binding domain of VEGFR-2, conbercept can bind to all isoforms of VEGF-A, VEGF-B, and placental growth factor, so it has a very strong effect on antiangiogenesis [110, 111]. KH902 has been widely used in China to treat ocular neovascular diseases which include wet AMD and diabetic retinopathy with great success and has been approved by FDA for stage III clinical trial [112–114]. This kind of VEGF inhibitor is much cheaper, while theoretically more efficient. However, conbercept has not been proven to treat CME secondary to BRVO or CRVO yet, but the phase III clinical trial has started in China recently.

Different anti-VEGF drugs may have varying treatment patterns and dosages. The recommended dosage is 0.5 mg, 2.0 mg, and 1.25 mg for ranibizumab, aflibercept, and bevacizumab, respectively [115, 116]. Some specialists preferred a monthly injection, while others employ a treat and extend (TREX) or an as needed *(pro re nata)* regimen [93, 106, 117–120]. A recent study reported that BRVO patients can receive good VA and central macular thickness (CMT) outcomes with a lower frequency of intravitreal ranibizumab. The total mean number of injections over 12 months follow-up was 2.1 for BRVO and 3.4 for CRVO [119]. In addition, frequent injection of VEGF inhibitors may also increase the risk of SAEs and could possibly lead to retinal atrophy secondary to obstruction of neuroprotective cytokines and regression of normal vasculature [121, 122]. There is still limited data on the comparative effectiveness of different anti-VEGF drugs at this time because the design of the studies varies from each other. Interestingly, bevacizumab is the preferred treatment option by 61% of the US retina specialists followed by aflibercept (17%) and ranibizumab (17%), as revealed by the American Society of Retina Specialists (ASRS) 2015 Membership Preferences and Trends Survey. This indicates that economical considerations contribute a major part toward decision making in clinical practice [123].

### 7.2. Corticosteroids

Intravitreal corticosteroids is another option, particularly in situations where the cost of treatment and the monthly treatment burden of anti-VEGF therapy is too difficult for patients [124]. The most commonly used steroid drug is triamcinolone acetonide (TA), which is reported to have similar or even better short-term outcomes especially in nonischemic BRVO compared with anti-anti-VEGF drugs. Research has demonstrated that both 3-month intravitreal injections of an anti-VEGF agent bevacizumab and two IVTA injections 2 months apart could be effective in respect to both visual and anatomical outcomes [125]. TA has much lower prices but has a higher rate of adverse events like increased intraocular pressure (IOP), cataract progression, and sterile pseudoendophthalmitis [101, 124, 126–130]. The SCORE study (*n* = 411) showed that the outcome of IVTA 1 mg groups is similar to IVTA 4 mg groups and can lower the rate of adverse events [131]. TA has numerous mechanisms of action, including anti-inflammatory effects, antiangiogenic properties and inhibition of VEGF, and other inflammatory cytokine expressions, such as IL-6, ICAM-1, and MCP-1 [132, 133]. Ozkiris et al. evaluated the effect of TA injection on persistent CME in BRVO that failed to respond to previous laser photocoagulation. During a mean follow-up time of 6.2 months, best-corrected VA improved significantly from 1.01 at baseline to 0.55 (LogMAR) at one month after the injection. VA after 3 months was 0.56, and at the end of follow-up was 0.62 [134]. Moreover, intravitreal TA (IVTA) may also improve macular sensitivity and morphology in patients with either ischemic or nonischemic BRVO [135].

Response to TA differs among eyes with edema, some only needing one injection, while most patients still need treatment for multiple times [136, 137]. Predictive factors for successful IVTA treatment were younger age, shorter duration of CME, initial onset CME, concurrent serous retinal detachment, few concomitant systemic diseases, intact foveal capillary ring, eyes with cystoid spaces in the outer plexiform layer, and nonischemic BRVO [134, 138].

Posterior subtenon triamcinolone acetonide (STA) has the advantage of easy injection and decreased risk of intraocular complications such as IOP elevation and cataract progression compared with IVTA, but the efficacy of STA is thought to be slightly less than that of IVTA [136, 139, 140].

Another steroid therapy is the dexamethasone intravitreal implant (Ozurdex), which provides continuous steroid delivery over a more sustained period, permitting longer duration of action and has been proved by previous studies [141–145]. The 12-month result of GENEVA trial (*n* = 1196) showed that eyes receiving DEX implant 0.7 or 0.35 mg achieved a 15-letter improvement in BCVA significantly faster than the eyes receiving sham treatment. At day 180, the cumulative response rate was 41% in the DEX implant 0.7 mg group, 40% in the DEX implant 0.35 mg group, and 23% in the sham group. There was no significant difference in efficacy and safety between 0.7 mg group and 0.35 mg group. However, the overall incidence of ocular adverse events was significantly higher in the DEX implant 0.7 mg group (62.9%) and DEX implant 0.35 mg group (61.9%) than in the sham group (42.8%; *P* < 0.001). The only adverse events that occurred significantly more frequently were eye pain (*P* = 0.023), ocular hypertension (*P* ≤ 0.002), and anterior chamber cells (*P* ≤ 0.031). Most of the participants met the visual acuity or retinal thickness criteria for retreatment at day 180 which showed as safe and well tolerated over 12 months. The safety and efficacy profile after a second treatment with DEX implant was generally similar to that seen after the first treatment, and patients who had delayed treatment never matched the improvement of those treated earlier in the disease process [146, 147]. The ORVO study (*n* = 17) also demonstrated that Ozurdex can reduce several propermeability proteins such as persephin, pentraxin 3, hepatocyte growth factor, endocrine gland VEGF, insulin-like growth factor binding proteins, and proinflammatory cytokines like MCP-1 and IL17-E [148, 149]. Also, the SHASTA study (*n* = 289) demonstrated that the visual acuity and central retinal thickness significantly improved both after initial or retreatment. 32.6% of patients were observed with elevated IOP whereas only 1.7% needed glaucoma surgery [150]. However, the OMAR study recently found no difference between Ozurdex and TA regarding anatomical or functional outcomes or the incidence of side effects, although the number of intravitreal injections was reduced by using Ozurdex [151]. Selected clinical trials are summarized in Table 2 [131, 146, 148, 150, 152]

### 7.3. Laser Photocoagulation

Venous occlusion is merely the initiating event that causes retinal ischemia and high levels of VEGF. The high levels of VEGF cause additional capillary closure and worsening ischemia, resulting in a positive feedback loop and disease progression over time in some patients [153, 154]. In 1986, the Branch Vein Occlusion Study (BRVOS) reported the efficacy of grid-pattern laser photocoagulation for treating macular edema due to BRVO and recommended this method as the standard treatment for BRVO [155]. The efficacy of grid-pattern laser photocoagulation is thought to result from changes in the biochemical processes within the retinal pigment epithelium (RPE) or hypoxia in the neural retina [156, 157]. The pigment epithelium-derived factor, which can inhibit retinal and choroidal neovascularization by inducing apoptosis in activated vascular endothelial cells, is reported to be upregulated in photocoagulated human retinal pigment epithelial cells [157, 158]. VEGF expression is found to increase a few days after laser treatment and then start to decrease [159]. Moreover, direct photocoagulation to leaking vessels and microaneurysms is found to be beneficial for treating chronic macular edema associated with chronic BRVO of longer than 12 months duration [160].

Peripheral scatter photocoagulation can reduce retinal ischemia. Theoretically, it may provide a way to interrupt the positive feedback loop in patients with BRVO and reduce the need for injections of a VEGF antagonist. However, the RELATE trial found that peripheral scatter laser did not benefit in BCVA, resolution of edema, or number of ranibizumab injections [92]. The group has also come up with three speculations: (1) the untreated area is hypoxic and can still release enough VEGF to cause persistent or recurrent edema; (2) chronic hypoxia, high levels of VEGF, and recurrent leakage can lead to structural changes in retina vessels; and (3) the reduction of VEGF is countered by inflammation and production of propermeability factors induced by photocoagulation [92].

Conventional laser therapy can result in enlarged retinal scars, subretinal fibrosis, choroidal neovascularization, and reduced macular sensitivity [161–164]. Subthreshold micropulse diode laser photocoagulation (SMDLP) is a less invasive treatment than conventional focal or grid laser therapy designed to produce lesions on the retinal pigment epithelium (RPE) while having minimal effect on the neurosensory retina. Small studies have demonstrated good clinical outcomes with micropulse laser, for initial visual acuity better or worse than 20/40, although larger studies are necessary [165–168]. However, in specific subsets of patients such as recurrent CME after conventional laser treatment, anti-VEGF treatment is still the preferred treatment rather than SMDLP [102].

Efficacy of conventional laser treatment is always limited, compared with anti-VEGF therapy and corticosteroids [90, 91, 169]. As a result, anti-VEGF therapy has taken the place of conventional laser treatment as first-line treatment for CME, and many studies put laser treatment as a rescue therapy or in combination therapy [97, 101, 170].

Another rarely used technique is laser-induced arteriolar construction (ACo), first described by L'Esperance in 1975. ACo is based on the sacrificial constriction of the afferent arteriole in the occluded BRVO region. Results using ACo have reported significantly improved BCVA in BRVO patients [171, 172]. Constriction of the afferent artery in the BRVO region accelerated the restoration of potassium channels and IL-6. These alterations may contribute to faster resorption of retinal edema and may decrease the level of inflammation [173]. However, further prospective randomized studies are needed.

### 7.4. Surgery

Considering the mechanism of vein occlusion, arteriovenous sheathotomy (AVS) would appear to be a reasonable treatment for BRVO. In 1988, Osterloh and Charles were the first to report a surgical procedure involving dissection of the common adventitial sheath at the level of the arteriovenous blockage site for decompressing the arteriovenous crossing [174]. The AVS not only released the pressure at A/V crossing but also decreased the IL-6 expression [175]. The efficacy of this method, however, was controversial [176–179]. A recent clinical trial using a modified control group demonstrated that eyes which went through AVS had significantly better vision acuity and central macular thickness than those in the control group, which is thought to be more convincing than the previous studies. But they also conducted internal limiting membrane peeling during the surgery, which may have some impact on the result [180]. Another study found that the pars plana vitrectomy (PPV) combined with AVS is safe and effective and can cause the disappearance of collateral vessels at the blockage site, which is an important clinical marker for intravascular reperfusion [181].

In 2004, Charbonnel et al. reported that eyes with an initial posterior vitreous detachment (PVD) had less improvement of visual acuity than those without a PVD after AVS and suggested that the surgical detachment of posterior hyaloid could be as important (or more) as the sheathotomy itself [182]. This theory was replicated by another study which showed that there was no significant difference in the improvement of macular function between the vitrectomy with or without arteriovenous adventitial sheathotomy group [183]. Similarly, vitrectomy with internal limiting membrane (ILM) peeling has been suggested as a potential treatment because it is generally believed that vitreous traction on the macula leads to fluid accumulation in the retina. Removal of posterior hyaloid may improve oxygenation of the retina [184]. However, Arai et al. found no difference in the results of vitrectomy with internal limiting membrane (ILM) peeling and that without ILM peeling, emphasizing the importance of vitrectomy [185]. Vitrectomy can help remove the vitreomacular traction and thus improve cytokines that affect vascular permeability. It was also reported that the oxygen tension is higher after vitrectomy, resulting in capillary shrinkage, reduced blood vessel leakage, and absorption of macular edema [186]. Posterior vitreous adhesion is thus considered an independent risk factor and also a prognostic factor for BRVO, and vitrectomy may be the most valuable part of the surgery [56, 75].

Another surgical method is the retinal bypass surgery, the feasibility has been proved recently this year, but the efficacy still needs further study [187]. Although surgery may not be the first choice to most physicians, it is still an option when other treatment is not effective in some patients [188].

### 7.5. Medical Treatment

It is reported that an increase in small platelet aggregates may play a component in BRVO pathogenesis. Beraprost and ticlopidine inhibit small aggregate formation in BRVO patients and may represent effective antiplatelet treatments [189]. Houtsmuller et al. compared the effect of ticlopidine, an antiplatelet aggregative factor, versus placebo in 54 patients with BRVO and found a significant improvement in visual acuity in 69% BRVO patients of ticlopidine group versus 52% of the placebo group in a six-month follow-up [190]. Glacet Bernard et al. examined the efficacy of troxerutin, an antierythrocyte and antiplatelet aggregative drug, versus placebo in 26 patients with BRVO less than five months from symptom onset. In a two-year follow-up, there was a significant improvement in visual acuity, as well as in macular edema, in patients treated with troxerutin compared to those treated with placebo [191].

Tissue plasminogen activator (t-PA) intravitreally or directly into the retinal vein is another treatment option for BRVO. Small studies have demonstrated the safety and an improvement in visual acuity and foveal thickness with t-PA treatment [192–196].

Low-molecular-weight heparins (LMWHs) have been also used and are considered to be effective for the treatment of BRVO, supporting the hypothesis that BRVO is a venous thrombotic disorder. No increased risk of vitreous hemorrhages was observed during treatment with LMWH while there was an improvement in the visual acuity [197, 198].

NSAIDs have been used to reduce the occurrence and severity of macular edema after cataract surgery, without causing elevation of intraocular pressure (IOP). A recent study with 15 BRVO patients suggested that intravitreal diclofenac is also safe and effective in improving BCVA and decreasing CMT in patients with BRVO and ME with the mean visual acuity improvement from 0.115 ± 0.03 preoperatively to 0.356 ± 0.29 (LogMAR). The mean preoperative CMT decreased from 453.2 *μ*m ± 55.3 *μ*m to 340.47 *μ*m ± 101 *μ*m at 3 months [199].

These medical therapies have demonstrated possible efficacy in treating CME due to BRVO in small studies. However, due to a small number of patients, further studies are necessary to ensure effectiveness.

### 7.6. Isovolemic Hemodilution

BRVO has been found to be associated with hyperviscosity due to higher hematocrit and plasma viscosity [22, 200]. Viscosity is mainly dependent upon the hematocrit and plasma fibrinogen. Higher blood viscosity is less important when blood flow rate is rapid. In conditions of low flow, as is likely in a vein predisposed to occlusion, the effect of viscosity becomes increasingly significant as a result of increased red cell aggregation. The enhanced aggregation at slow flow rates further decreases flow leading to a vicious cycle of increased viscosity promoting increased aggregation which further increases viscosity, resulting in a state of “rheological obstruction” [22, 201]. Additionally, the occlusion-induced hypoxia will increase blood viscosity as acidosis increases red cell aggregability and reduces red cell deformability [202, 203]. And both these red cell anomalies may pre-exist in patients with BRVO [204]. A study investigated 34 BRVO patients and obtained positive effect on the visual outcome with a target hematocrit of 35%. The visual acuity in the isovolemic hemodilution therapy group improved by 0.20 LogMAR units at 6 weeks and 0.43 −LogMAR units at 1 year, which was statistically significant compared with that in the control group [23]. However, the systemic complications of this method make it less desirable than other available therapies.

### 7.7. Combined Therapy

There are many forms of treatment to BRVO as described above. Therefore, some investigators came up with the idea that combination therapy with two or more agents may be beneficial to get better outcomes with lowered dose, rate of adverse events, and less frequent treatment. Repeated intravitreal injections of an anti-VEGF can cause adverse effects such as ocular pain, ischemic retinopathy, and endophthalmitis, in addition to the high cost of anti-VEGF drugs like ranibizumab [205, 206]. It is reported that bevacizumab combined with macular grid and scatter laser photocoagulation targeted retinal photocoagulation (TRP) of peripheral nonperfused areas (NPAs) could significantly improve vision, reduce macular edema, and prevent the recurrence of CME better than bevacizumab alone [207, 208]. Also, a lower number of reinjections were observed in the combined treatment [209]. The application of topical bromfenac, a nonsteroidal anti-inflammatory drug during IVB therapy in eyes with ME secondary to BRVO, was found to have the advantage of reducing the number of injections although it did not affect the visual prognosis [210]. Other combination therapies that have been investigated include corticosteroids with laser, anti-VEGF agents with corticosteroids, anti-VEGF agents with laser, and AVS or PPV with corticosteroids, to name a few [211–219]. Although most of these studies reported excellent outcomes, it is difficult to compare between studies to discern which is the best combination.

## 15. Conclusion

Branch retinal vein occlusion is a very common retinal disease. Numerous studies have been carried out evaluating every aspect of this ocular vascular dysfunction. The most commonly used treatments at this time are anti-VEGF drugs and corticosteroids, since they bring about significant improvement in VA and CMT with relatively fewer complications. Even if one treatment is not effective in particular patients, there are numerous treatments and combination therapies that can be considered. Novel treatments have revolutionized our care of patients with retinal vein occlusions. Further studies into the pathophysiology, risk factors, and treatment of retinal vein occlusions remain ongoing, and we continue to improve our treatment of patients with this difficult disease through personalized medicine and development of new methods and treatments.

## Figures and Tables

**Table 1:** Clinical trials evaluating intravitreal anti-VEGF agent for macular edema due to BRVO.

Study	Author/year	Method	Duration	Subjects	Mean BCVA change	Mean CMT change (*μ*m)	Mean number of injections
BERVOLT	Kornhauser et al. 2016	Bevacizumab 0.05 ml	24 months	87	+0.25 (LogMAR)	−193.9	7.6

BRIGHTER	Tdayoni et al. 2016	Ranibizumab 0.5 mg	24 months	183	+14.8 (ETDRS)	223.3	4.8
		Ranibizumab 0.5 mg + laser		180	+14.8	−240.1	4.5
		Laser alone (3 + PRN)		92	+6.0 (6 months)	−89 (6 months)	N/A

VIBRANT	Clark et al. 2016	Aflibercept 2.0 mg	52 weeks	91	+17.1 (ETDRS)	−283.9	9.0
		(6 + 1 per 2 months) Grid laser		92	+12.2	−249.3	N/A

RELATE	Campochiaro et al. 2015	Ranibizumab 2.0 mg	144 weeks	20	+14.6 (ETDRS)	−292.1	6
		Ranibizumab 0.5 mg		22	+12.1	−203.3	6
		Second randomization after 24 weeks:	
		Ranibizumab		16	+3.1	+36.6	14.9
		Ranibizumab + laser (6 + PRN)		17	−2.6 (from week 24 to week 144)	−3.2	15.6

MARVEL	Raja et al. 2015	Ranibizumab 0.5 mg	6 months	37	+18.1 (ETDRS)	−177.1	3.2
		Bevacizumab 1.25 mg (PRN)		38	+15.6	−201.7	3.0

RABAMES	Pielen et al. [94]	Ranibizumab 0.5 mg	6 months	10	+17 (ETDRS)	+142.4	3
		Ranibizumab 0.5 mg + laser		10	+6	+171.7	3
		Laser only (monthly)		10	+2	−37.6	N/A

COMRADE-B	Hattenbach et al.	Ranibizumab 0.5 mg (3 + PRN)	6 months	126	+14.15 (ETDRS)	−275	4.7
		Dexamethasone 0.7 mg		118	+9.66	−130	1

BRAVO	Brown et al. [95]	Ranibizumab 0.3 mg	12 months	134	+16.4 (ETDRS)	−313.6	8.3
		Ranibizumab 0.5 mg		131	+18.3	−347.4	8.4
		Sham/ranibizumab 0.5 mg (6 + PRN)		132	+12.1	−273.7	5.7

HORIZON (12-month open-label extension of BRAVO trial)	Heier et al. [96]	Ranibizumab 0.3/0.5 mg	12 months	103	+0.9 (ETDRS)	+3.7	2.4
		Ranibizumab 0.5/0.5 mg		104	−2.3	+6.3	2.1
		Sham/ranibizumab 0.5 mg (PRN)		97	−0.7	+35.3	2

RETAIN (Prospective follow-up of a subset of patients from HORIZON study)	Campochiaro et al. 2014	Ranibizumab 0.5 mg (PRN)	49 months	34	2 years:	2 years: −7.2	2 years: 2.6
					−0.4 (ETDRS)		
					3 years: +2.6	3 years: −42.5	3 years: 2.1
					4 years: +0.5	4 years: −26.2	4 years: 2.0
					(Compared with year 1**)**		

SHORE	Campochiaro et al. 2014	Ranibizumab 0.5 mg PRN	15 months	50	+21 (ETDRS)	−247.8	3.8
		Ranibizumab 0.5 mg (7 + PRN)		48	+18.7	−289.9	7.6

COMO	Francesco et al.	Ranibizumab 0.5 mg (6 + PRN)	12 months	~200	N/A	N/A	N/A
		Dexamethasone 0.7 mg		~200	N/A	N/A	N/A

Note: only BRVO patients are analyzed in this table. Outcomes are not directly comparable because study design and populations varied from each other. The COMO trial is still undergoing; therefore, there are no results available at this time. BRVO: branch retinal vein occlusion; VEGF: vascular endothelial growth factor; BCVA: best-corrected visual acuity; CMT: central macular thickness; ETDRS: early treatment diabetic retinopathy study; LogMAR: logarithm of the minimum angle of resolution; BERVOLT: bevacizumab for RVO long-term follow-up; BRIGHTER: individualized stabilization criteria-driven ranibizumab versus laser in branch retinal vein occlusion; VIBRANT: intravitreal aflibercept for macular edema following branch retinal vein occlusion; RELATE: scatter photocoagulation does not reduce macular edema or treatment burden in patients with retinal vein occlusion; MARVEL: a randomized, double-masked, controlled study of the efficacy and safety of intravitreal bevacizumab versus ranibizumab in the treatment of macular edema due to branch retinal vein occlusion; RABAMES: ranibizumab for branch retinal vein occlusion associated macular edema study; BRAVO: the ranibizumab for the treatment of macular edema following branch retinal vein occlusion; HORIZON: ranibizumab for macular edema due to retinal vein occlusions; RETAIN: long-term outcomes in patients with retinal vein occlusion treated with ranibizumab; SHORE: study evaluating dosing regimens for treatment with intravitreal ranibizumab injections in subjects with macular edema following retinal vein occlusion; COMO: comparison of intravitreal dexamethasone implant and ranibizumab for macular edema in BRVO.

**Table 2:** Clinical trials evaluating intravitreal corticosteroids for macular edema due to BRVO.

Study	Author/year	Method	Duration	Subjects	Mean BCVA change	Mean CMT change (*μ*m)	Number of injections
ORVO	Campochiaro et al. 2015	Dexamethasone 0.7 mg (all patients have treated with anti-VIGF agents before)	16 weeks	17	4 weeks: ~+5.8 (ETDRS) 16 weeks: ~+5.8 (Peak at 4 weeks)	4 weeks: ~−153 16 weeks: ~−60 (Peak at 4 weeks)	1

SHASTA	Capone et al. [150]	Dexamethasone 0.7 mg	1.2 years	157	~+5.0 (ETDRS)	−154~−188	3.2

SOLO	Bezatis et al. [152]	Dexamethasone 0.7 mg	6 months	54	8 weeks: +0.3 (LogMAR) 24 weeks: +0.15 (Peak at 8 weeks)	8 weeks: −214 24 weeks: −107 (Peak at 8 weeks)	1.3

GENEVA	Haller et al. [146]	Dexamethasone 0.35 mg/0.7 mg		208	60 days: ~+10.2 (ETDRS) 180 days: ~+6.1	180 days: −103 360 days: −163	1.85
		Dexamethasone 0.7 mg/0.7 mg	12 months	227	60 days: ~+10.1 180 days: ~+6.2 360 days: ~+6.3	180 days: −97 360 days: −166	1.86
		Sham/0.7 mg (0–6 months/6–12 months)		210	60 days: ~+4.7 180 days: ~+3.8 360 days: ~+6.1 (Peak at 60 days)	180 days: −102 360 days: −170	0.83

SCORE	Ingrid et al. 2009	Triamcinolone 1 mg Triamcinolone 4 mg Standard care	12 months	136 137 137	+5.7 (ETDRS) +4 +4.2	−245 −250 −312	2.2 2.1 N/A

Note: only BRVO patients are analyzed in this table. Outcomes are not directly comparable because study design and populations varied from each other. Some studies did not report the accurate number and are estimated by the figures. BRVO: branch retinal vein occlusion; BCVA: best-corrected visual acuity; CMT: central macular thickness; ETDRS: early treatment diabetic retinopathy study; LogMAR: logarithm of the minimum angle of resolution; ORVO: the Ozurdex for retinal vein occlusion study: SHASTA: efficacy and safety of two or more dexamethasone intravitreal implant injections for treatment of macular edema related to retinal vein occlusion; SOLO: functional and anatomical results after a single intravitreal Ozurdex injection in retinal vein occlusion; GENEVA: sham-controlled randomized trial of dexamethasone intravitreal implant in patients with macular edema due to retinal vein occlusion; SCORE: the standard care versus corticosteroid for retinal vein occlusion trial.
